# Comprehensive Analysis of DNA Methylation in Head and Neck Squamous Cell Carcinoma Indicates Differences by Survival and Clinicopathologic Characteristics

**DOI:** 10.1371/journal.pone.0054742

**Published:** 2013-01-24

**Authors:** Justin A. Colacino, Dana C. Dolinoy, Sonia A. Duffy, Maureen A. Sartor, Douglas B. Chepeha, Carol R. Bradford, Jonathan B. McHugh, Divya A. Patel, Shama Virani, Heather M. Walline, Emily Bellile, Jeffrey E. Terrell, Jay A. Stoerker, Jeremy M. G. Taylor, Thomas E. Carey, Gregory T. Wolf, Laura S. Rozek

**Affiliations:** 1 Department of Environmental Health Sciences, University of Michigan School of Public Health, Ann Arbor, Michigan, United States of America; 2 School of Nursing, University of Michigan, Ann Arbor, Michigan, United States of America; 3 Department of Otolaryngology, University of Michigan Medical School, Ann Arbor, Michigan, United States of America; 4 Department of Computational Medicine and Bioinformatics, University of Michigan, Ann Arbor, Michigan, United States of America; 5 Department of Pathology, University of Michigan Medical School, Ann Arbor, Michigan, United States of America; 6 Department of Obstetrics, Gynecology and Reproductive Sciences, Yale University, New Haven, Connecticut, United States of America; 7 Comprehensive Cancer Center, University of Michigan, Ann Arbor, Michigan, United States of America; 8 Sequenom Center for Molecular Medicine, San Diego, California, United States of America; 9 Department of Biostatistics, University of Michigan School of Public Health, Ann Arbor, Michigan, United States of America; Karolinska Institutet, Sweden

## Abstract

Head and neck squamous cell carcinoma (HNSCC) is the eighth most commonly diagnosed cancer in the United States. The risk of developing HNSCC increases with exposure to tobacco, alcohol and infection with human papilloma virus (HPV). HPV-associated HNSCCs have a distinct risk profile and improved prognosis compared to cancers associated with tobacco and alcohol exposure. Epigenetic changes are an important mechanism in carcinogenic progression, but how these changes differ between viral- and chemical-induced cancers remains unknown. CpG methylation at 1505 CpG sites across 807 genes in 68 well-annotated HNSCC tumor samples from the University of Michigan Head and Neck SPORE patient population were quantified using the Illumina Goldengate Methylation Cancer Panel. Unsupervised hierarchical clustering based on methylation identified 6 distinct tumor clusters, which significantly differed by age, HPV status, and three year survival. Weighted linear modeling was used to identify differentially methylated genes based on epidemiological characteristics. Consistent with previous *in vitro* findings by our group, methylation of sites in the *CCNA1* promoter was found to be higher in HPV(+) tumors, which was validated in an additional sample set of 128 tumors. After adjusting for cancer site, stage, age, gender, alcohol consumption, and smoking status, HPV status was found to be a significant predictor for DNA methylation at an additional 11 genes, including *CASP8* and *SYBL1*. These findings provide insight into the epigenetic regulation of viral vs. chemical carcinogenesis and could provide novel targets for development of individualized therapeutic and prevention regimens based on environmental exposures.

## Introduction

Head and neck squamous cell carcinomas (HNSCCs), the eighth most commonly diagnosed cancer in the U.S. population, have a complex etiology that includes life style behaviors, classical chemical carcinogenesis, and infection with high risk types of human papillomavirus (HPV). Traditionally, head and neck cancer is associated with a profound history of tobacco and alcohol use, and poor survival compared to other cancers [1]. Over the last decade, high-risk HPV has emerged as a risk factor for head and neck cancer, particularly of the oropharynx [2], [3]. Patients with HPV(+) head and neck cancer have a distinct risk profile, associated with a less remarkable history of tobacco and alcohol use [4], a more beneficial micronutrient profile [5], and improved survival compared to those with HPV(−) tumors [6].

Both tobacco- and alcohol-related, as well as HPV-associated, head and neck cancers have a well-described multistep model of carcinogenesis [7]. Broadly, mutations or loss of heterozygosity of major cell cycle regulator genes, such as *p53*, are frequently detected in tobacco and alcohol-related head and neck cancers [8], [9], although mutation at these genes has not consistently been associated with patient survival. Likewise, HPV(+) head and neck cancers are associated with functional inactivation of *p53* and *Rb*, which is mediated by E6 and E7 viral oncoproteins, resulting in overexpression of *p16* [10], [11], [12]. Conversely, HPV(+) head and neck cancers have a distinct clinical profile when compared to alcohol and tobacco-related HPV(−) tumors, the former of which are typically more responsive to treatment [13].

Epigenetic modifications are an important mechanism in carcinogenic progression [14], but the epigenetic profiles between HPV(+) and HPV(−) tumors remain poorly characterized, with most studies focusing on specific loci or global markers of DNA methylation [15], [16]. A handful of epigenome-wide studies of head and neck cancer have focused on differences between normal and tumor tissue, associations with alcohol and tobacco exposure, and associations with global marks of DNA methylation [17], [18].

Recently, we reported an epigenome-wide analysis of concurrently measured DNA methylation and gene expression in HPV(+) and HPV(**−**) squamous cell carcinoma cell lines, noting that HPV(+) cell lines have higher amounts of genic methylation as well as increased expression of *DNMT3A* [19]. Information about the specific epigenome-wide differences in DNA methylation based on clinical characteristics, including HPV infection, remain unknown, and require a well-characterized cohort of patient samples. In this study, a comprehensive methylation bead array was used to measure DNA methylation at 1505 CpG sites across 807 genes in both HPV(−) and HPV(+) head and neck cancer in tumor samples collected from the ongoing patient cohort in the University of Michigan Head and Neck Specialized Program of Research Excellence (SPORE). In addition, important survival differences by epigenetic profiles are identified as described. Findings from this study provide insight into the epigenetic regulation of viral vs. chemical carcinogenesis and provide novel targets for development of individualized therapeutic regimens based on environmental exposures.

## Methods

### Design

Subjects for this study were obtained from a prospective, cohort study of patients enrolled in the University of Michigan Head and Neck Cancer SPORE. Newly diagnosed patients were recruited, provided informed consent, and followed quarterly for 2 years and then yearly thereafter. In addition tumor samples were collected. Institutional Review Board approval was approved from all participating sites including the Institutional Review Boards of the University of Michigan Medical School and the Institutional Review Board for Human Subject Research at the Veterans Affairs Ann Arbor Healthcare System.

#### Study population

Individuals eligible for participation included patients diagnosed with first primary head and neck cancer between January 1, 2003 and December 31, 2005 completed an epidemiologic questionnaire, and had paraffin-embedded tumors available for analysis with adequate residual tissue for microdissection (N = 82). The epidemiologic questionnaire included questions about lifestyle behaviors, including smoking and drinking. Clinical characteristics included tumor site and stage, comorbidities, depression, quality of life, and recurrence status, as well as treatment modalities. Tumor blocks were re**-**cut for uniform histopathologic review and microdissection, with the first and last slides in a series of 12 reviewed by a qualified pathologist (JM) to confirm the original diagnosis and to circle areas for DNA extraction. Percent cellularity was estimated for each tumor and areas with >70% cellularity of cancer were designated for use in the analyses.

### Laboratory Methods

#### FFPE tissue, DNA isolation, bisulfite conversion

Regions identified for DNA extraction were cored from the formalin fixed paraffin embedded (FFPE) tissue blocks using an 18 gauge needle. Isolation of DNA from cored tissue samples was performed using the QIAamp DNA FFPE Tissue Kit (Qiagen, Valencia, CA) modified to include overnight incubation at 56°C in lysis buffer. DNA concentration and purity were confirmed via NanoDrop spectrophotometer (Thermo Scientific, Waltham, MA). Sodium bisulfite modification was performed on 500 ng to 1 µg of extracted DNA using the EZ DNA Methylation kit (Zymo Research, Orange, CA) following the manufacturer’s recommended protocol.

#### HPV testing

HPV status was determined by an ultra-sensitive method using real-time competitive polymerase chain reaction and matrix-assisted laser desorption/ionization-time of flight mass spectroscopy with separation of products on a matrix-loaded silicon chip array, as described in Tang et al. [20]. Multiplex PCR amplification of the E6 region of 15 discrete high-risk HPV types (HPV 16, 18, 31, 33, 35, 39, 45, 51, 52, 56, 58, 59, 66, 68 and 73), and human GAPDH control was run to saturation followed by shrimp alkaline phosphatase quenching. Amplification reactions included a competitor oligo identical to each natural amplicon except for a single nucleotide difference. Probes that identify unique sequences in the oncogenic E6 region of each type were used in multiplex single base extension reactions extending at the single base difference between wild-type and competitor HPV so that each HPV type and its competitor were distinguished by mass when analyzed on the MALDI-TOF mass spectrometer as described previously [21], [22], [23], [24].

#### Bead array methods

The commercially available Illumina Goldengate® Methylation Cancer Panel was used to detect DNA methylation patterns in tumor samples. The Cancer Panel measure DNA methylation at 1505 CpG sites located in known CpG islands across 807 genes related to cancer, including oncogenes, tumor suppressor genes, imprinted genes, and genes involved in cell cycle regulation, DNA repair, apoptosis and metastasis. Bead arrays were processed at the University of Michigan DNA Sequencing Core Facility according to the manufacturer’s protocol. Briefly, bisulfite converted tumor DNA was hybridized to the bead array as described previously [25], and bead arrays were imaged using Illumina BeadArray Reader software. Raw bead array fluorescence data were initially analyzed using Illumina BeadStudio Methylation software, which converts fluorescence values of the methylated (Cy5) and unmethylated (Cy3) alleles into an average methylation value at a specific probe using the formula β = [Max(Cy5,0)]/[Max(Cy5,0)+Max(Cy3,0) +100], ranging from completely unmethylated (β = 0) to completely methylated (β ≈ 1). For each probe, background fluorescence, as estimated from a set of negative controls, was subtracted. Fourteen of the 82 samples (17.1%) failed on the array were excluded from further analyses, resulting in a final sample size of 68 tumors.

Methylation at specific CpG probes on the Goldengate BeadArray has been shown to be biased by probe thermodynamic properties [26]. Known biases include probe length and GC content, which can affect the melting temperature of the probes as well as probe fluorescence intensities. Thus, we used the method proposed by Kuan et al. to normalize our average β values based on probe length and GC content [26].

Detection p-values on the Goldengate BeadArray are calculated based on fluorescence signal at a probe compared to background fluorescence and represent the (1-probability) that a signal is stronger than background fluorescence. The weighted methodology proposed by Kuan et al. was used to develop sample and site weights based on p-values of detection. Both samples and sites with larger detection p-values are generally considered less reliable and were down-weighted in further gene specific analyses.

#### Site specific validation

DNA methylation of four CpG sites in the promoter region of *CCNA1* was quantified in an additional sample of 128 pretreatment head and neck tumors using the Sequenom EpiTyper, a MALDI-TOF mass spectrometry based platform. DNA was extracted from FFPE tumors, HPV status was determined, and the DNA was bisulfite converted as described above. Bisulfite PCR amplification was performed using FastStart *Taq* Polymerase (Roche Diagnostics, Indiana, US) with a forward and reverse primer concentration of 0.2 µM and an annealing temperature of 48C and 45 PCR cycles. The primer sequences, including the forward and T7 promoter tags required for Sequenom analysis were: 5′-AGGAAGAGAGATGTATTTTGGATTTTTTATTGGGG (forward primer) and 5′-CAGTAATACGACTCACTATAGGGAGAAGGCTAAAAAAACATTCTAACAAACCTCCA (reverse primer). Methylation analyses were performed at the University of Michigan Sequencing Core Facility following the manufacturer’s recommended protocol.

### Statistical Methods

Unsupervised hierarchical clustering was performed using the Euclidean distance metric and the Ward clustering method in the *hclust* package in R version 2.10.1. [27]. All 68 tumor samples were included in the hierarchical clustering algorithm. To minimize sex-specific effects, we excluded CpG sites on the sex chromosomes. The cluster analysis was performed using three different cutoffs for inclusion of individual CpG sites; the 50%, 25%, and 10% of CpG sites with the highest variance in methylation across samples.

Clinical characteristics were evaluated across clusters based on cluster membership using non-parametric rank-based and exact statistics. For survival analyses, death was considered an “event”; survival time was censored at 3 years (1095 days). The Kaplan-Meier method was used to estimate overall survival and the log-rank test was used to test differences in survival distributions using the R *survival* package. Differences in age were compared using analysis of variance (ANOVA). Fisher’s Exact test was performed to test differences in cancer site, stage, and HPV status. Cox proportional hazards models were constructed to test the association between methylation at each CpG site on the Goldengate BeadArray and survival, adjusting for HPV status, gender, age, disease stage, cancer site, smoking status, and problem drinking using the *coxph* function in the R *survival* package. Individuals with a tumor testing positive for any strain of HPV were considered HPV positive. Age was treated as a continuous variable, while disease stage and cancer site were treated as categorical variables. Smoking was categorized into never smoker, past former smoker (quit more than 12 months ago), recent former smoker (quit in previous 12 months), and current smoker. Problem drinking was defined as a score of greater than 8 on the validated Alcohol Use Disorders Identification Test, as previously described [28]. Due to the simultaneous testing of multiple proportional hazard models, we controlled the false discovery rate by calculating the false discovery rate q-value [29]. Q-values were calculated using the *qvalue* R package.

Overall site specific methylation differences between HPV(+) and HPV(−) tumors were compared by calculating the difference in the mean methylation per CpG site in HPV(+) and HPV(−) tumors. The effects of clinical characteristics on DNA CpG methylation measured on the Goldengate array were examined using *Limma* in R 2.10.1 [30]. Sample weights generated with *LumiWCluster* based on detection p-values across samples were used in the *lmFit* function from the *Limma* package to downweight samples with higher detection p-values. CpG sites were identified as differentially methylated between HPV(+) and HPV(−)tumors, adjusting for cancer site (oral cavity, nasopharynx, oropharynx, hypopharynx, larynx), cancer stage, sex and age. An empirical Bayes method (using the *eBayes* function in *Limma*) was used to rank CpG sites in order of significance of differential methylation. Additionally, *Limma* was used to examine methylation differences between the case cluster with significantly worse survival compared to the remaining cases. For CCNA1 validation, mean methylation was calculated across the 4 sites measured by the EpiTyper and compared across HPV(+) and HPV(−) tumors using the Wilcoxon rank-sum test. Additionally, a multiple linear regression model was constructed with mean CCNA1 methylation as the independent variable and HPV status as the main predictor, adjusting for age, sex, tumor site, and tumor stage. Gene Set Enrichment Analysis (GSEA) was used to identify common pathways and chromosomal locations for genes identified as significant (p<0.05) in the *Limma* analysis [31]. Statistically significant genes were ranked by t-value and input into GSEA as a ranked list. The full list of genes assayed on the Goldengate BeadArray were input into GSEA as a chip platform file, which provided the background for the enrichment analysis. Weighted enrichment statistics were calculated by the GSEA software, using a minimum analyzed gene set size of 5.

## Results

### Descriptive Statistics: Study Samples

The mean age of the 68 subjects was 57 years (range: 28–82 years)**;** 75% of the subjects were male. The majority of the HNSCCs were from the oropharynx (47%), oral cavity (25%), and larynx (19%), with a large proportion of cancers diagnosed as late stage (22% stage III and 62% stage IV, **Table 1**). Approximately one-third of the tumors tested positive for HPV (20 HPV-16, 2 HPV-18, 1 HPV-35 and 1 HPV-59). The majority of the patients were former (60%) or current (24%) cigarette smokers and 34% screen positive for problem drinking. All patients were treated in a standardized fashion with single modality treatment for patients with early stage tumors (Stage I/II) and combined chemotherapy and radiation and in some cases surgery for patients with advanced (Stage III/IV) cancers. Median follow up for the entire cohort was 60 months (95% CI: 59.9, 60.0).

**Table 1:** Clinical characteristics of the study participants (n = 68).

Patient Characteristic		N (%)	Mean (SD), Median (range)
Age			57.0(10.0), 55.0 (28–82)
Gender	Male	51 (75%)	
	Female	17 (25%)	
Stage	I and II	11 (16%)	
	III	15 (22%)	
	IV	42 (62%)	
Cancer Site of first Primary	Oral Cavity	17 (25%)	
	Oropharynx	32 (47%)	
	Hypopharynx	4 (6%)	
	Larynx	13 (19%)	
	Other	2 (3%)	
Tumor Tissue HPV (+) Status		24 (35%)	
Smoking Status	Never	11 (16%)	
	Past	41 (60%)	
	Current	16 (24%)	
Pack-years			33.3(37), 25 (0–220)
Non-cigarette Tobacco	(yes/no) ever	12 (18%)	
Alcohol Problem	AUDIT > = 8 and drank within 1 year	23 (34%)	

### General Clustering: Cluster Characteristics

**Figure 1:**
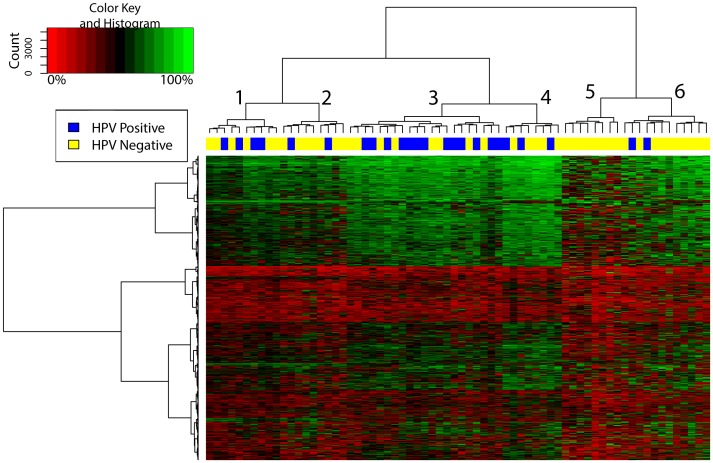
DNA methylation heatmap constructed using unsupervised hierarchical Ward clustering of the 711 CpG sites with the greatest variance across the 68 tumor samples identified six distinct methylation clusters.

Excluding CpG sites located on the sex chromosomes (n = 84), and limiting the cluster analysis to the 50% of CpG sites with the most variance (n = 711), six distinct clusters were identified (**Figure 1** **)**. Clusters by epidemiological and clinical characteristics were assessed first. Individuals who grouped in Cluster 3 tended to be older (mean age = 61.6 years, **Table 2**) and were significantly more likely to be HPV positive (62%, p = 0.02). There was no significant difference in the proportion of individuals who were problem drinkers in each of the clusters. Tumor samples from individuals grouped into Cluster 5 were more likely to have widespread DNA hypomethylation, while tumor tissue from individuals in Clusters 3 and 4 tended to have higher levels of methylation in the most differentially methylated genes. A similar distribution of epidemiological characteristics was observed across clusters when including only the 25% of CpG sites with the greatest variance, which revealed 3 distinct clusters, with HPV (p = 0.004) and age (p = 0.04) remaining statistically significantly different. These differences were not observed when restricting the analysis to only the top 10% most variable CpG sites, where 4 distinct tumor clusters were observed, and neither age (p = 0.41) nor HPV (p = 0.07) were statistically significantly different across clusters. There was no clear clustering of the tumors from the HPV-18, HPV-35, or HPV-59 individuals.

**Table 2:** Clinical characteristics of the six clusters identified via unsupervised hierarchical cluster analysis of DNA methylation values.

	Cluster 1	Cluster 2	Cluster 3	Cluster 4	Cluster 5	Cluster 6
N	10	9	21	9	8	11
Male n (%)	7 (70%)	9 (100%)	16 (76%)	6 (67%)	5 (63%)	8 (73%)
Age in years						
Mean (sd)	50.7 (9.1)	55.4 (8.2)	61.6 (9.7)	51.8 (6.4)	57.9 (9.6)	58.9 (11.9)
Median (min-max)	51.5 (28–61)	54 (42–68)	62 (41–82)	53 (43–64)	57.5 (46–72)	64 (41–73)
Cancer Site n (%)						
OC	2 (20%)	1 (11%)	1 (5%)	3 (33%)	5 (63%)	5 (45%)
OP	6 (60%)	4 (44%)	15 (71%)	4 (44%)	0	3 (27%)
HP	1 (10%)	1 (11%)	1 (5%)	0	0	1 (9%)
LA	1 (10%)	2 (22%)	4 (19%)	2 (22%)	3 (37%)	1 (9%)
OT	0	1 (11%)	0	0	0	1 (9%)
Primary Cancer Stage n (%)						
I and II	0	0	3 (14%)	1 (11%)	3 (38%)	4 (36%)
III	4 (40%)	3 (33%)	2 (10%)	2 (22%)	2 (25%)	2 (18%)
IV	6 (60%)	6 (67%)	16 (76%)	6 (67%)	3 (38%)	5 (45%)
HPV status n (%)*						
Pos	4 (40%)	1 (11%)	13 (62%)	3 (33%)	0	2 (18%)
Neg	6 (60%)	8 (89%)	8 (38%)	6 (67%)	8 (100%)	9 (82%)
Smoking Status n (%)						
Currently smoke cigarettes	1 (10%)	3 (33%)	5 (24%)	4 (44%)	0	3 (27%)
Past smoker, quit within last year	5 (50%)	4 (44%)	3 (14%)	3 (33%)	5 (63%)	4 (36%)
Past smoker, quit over a year ago	3 (30%)	1 (11%)	7 (33%)	0	3 (38%)	3 (27%)
Never smoked cigarettes	1 (10%)	1 (11%)	6 (29%)	2 (22%)	0	1 (9%)
Problem Drinking n (%)^a^	4 (40%)	5 (56%)	5 (24%)	3 (33%)	3 (38%)	3 (27%)
3 year Overall Survival*	7 (70%)	6 (66%)	18 (86%)	3 (66%)	2 (25%)	9 (82%)
Treatment						
Surgery Only	1 (10%)	0	4 (19%)	1 (11%)	2 (25%)	0
Radiation Only	0	1 (11%)	1 (5%)	0	0	1 (9%)
Surgery and Radiation	0	2 (22%)	3 (14%)	2 (22%)	0	2 (18%)
Radiation and Chemotherapy	4 (40%)	4 (44%)	8 (38%)	4 (44%)	4 (50%)	7 (64%)
Surgery, Radiation and Chemotherapy	5 (50%)	2 (22%)	3 (14%)	2 (22%)	0	2 (18%)

* p<0.05 for difference between clusters.

a Problem drinking defined: AUDIT>8 and drank in past 1 year. Note: n = 14 missing AUDIT score.

**Figure 2:**
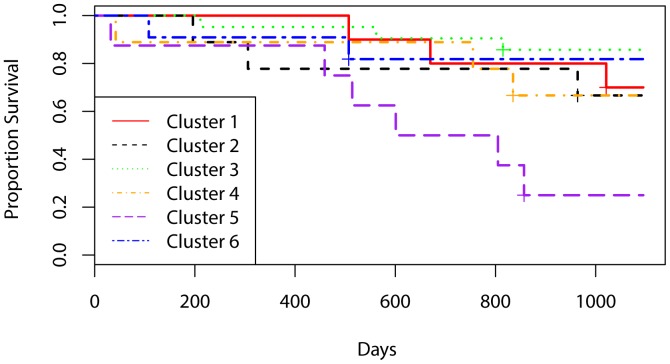
Kaplan-Meier survival curves depicting three year survival for each of the six clusters identified via unsupervised hierarchical cluster analysis.

Next, cluster membership was characterized by survival. Three year survival was compared between the six clusters (**Figure 2**). Overall, individuals in Cluster 3 had the best three year survival (86%) while individuals in Cluster 5 had the worst overall survival (25%). Cluster membership was found to be a significant predictor of three year survival (p = 0.02). HPV(+) cases were found to have statistically significant better three year survival than HPV(−) cases (p = 0.03). Interestingly, Cluster 3 had the highest proportion of Stage IV disease, the highest proportion of HPV(+) tumors, and the best three-year survival, while Cluster 5 had the lowest proportion of Stage IV disease and the worst survival. This aligns with previous findings that HPV positive tumors have a better prognosis, leading to the increased survival rates observed for Cluster 3 [13]. This also aligns with the observation that many HPV-positive patients present with advanced nodal disease.

### CpG Site-Specific Methylation Differences by HPV Status

Plotting average differences in methylation at each site showed that HPV(+) tumors tended to be hypermethylated at more sites than HPV(−) tumors (**Figure S1**). In order to better understand how HPV infection affects the DNA methylation profile in head and neck cancer, associations between methylation at each of the 1505 CpG sites on the Goldengate array and HPV status were calculated. Thirteen individual CpG sites on the array were found to be significantly associated with the HPV status of the tumor with a q-value <0.05 (**Table 3**)**.** The top hit, a CpG site located slightly downstream of the transcription start site of *CCNA1* in a CpG island, was found to be significantly more methylated in HPV(+) tumors (p = 1.8×10^−6^). This finding corroborates our recent analysis of epigenome-wide DNA methylation differences in HPV(+) and HPV(−) cell lines where *CCNA1* was found to be a major interaction hub following bioinformatic analyses [19]. CpG sites in *GRB7*, *CDH11*, *RUNX1T1, SYBL1,* and *TUSC3* were also found to be significantly more methylated in HPV(+) tumors. CpG sites in *SPDEF, RASSF1, STAT5A, MGMT, ESR2, JAK3,* and *HSD17B12* were found to be significantly hypomethylated in HPV(+) tumors (**Table S1)**.

**Table 3:** CpG sites with DNA methylation values significantly associated (Adjusted p<0.05) with HPV status of the tumor.

Gene Symbol	Chromosome	CpGCoordinate	Distanceto TSS	DNA Strand of Transcription	T-Value	Mean % Difference in Methylation	P-Value	AdjustedP-Value
CCNA1	13	35904640	7	+	5.30	21.3	1.86E-06	0.0028
GRB7	17	35147553	−160	−	4.58	8.0	2.46E-05	0.0161
SPDEF	6	34631953	116	−	−4.51	−3.7	3.20E-05	0.0161
CDH11	16	63713774	−354	−	4.32	18.4	6.08E-05	0.0192
RUNX1T1	8	93176474	145	−	4.31	13.7	6.37E-05	0.0192
RASSF1	3	50353615	−244	+	−4.22	−2.1	8.47E-05	0.0213
STAT5A	17	37693133	42	+	−4.05	−11.5	1.51E-04	0.0318
MGMT	10	131155184	−272	−	−4.01	−3.6	1.73E-04	0.0318
ESR2	14	63830765	66	+	−3.98	−6.4	1.90E-04	0.0318
JAK3	19	17819736	64	+	−3.92	−11.8	2.31E-04	0.0348
SYBL1	X	154763858	−349	+	3.88	12.2	2.71E-04	0.0370
HSD17B12	11	43659026	145	−	−3.83	−0.9	3.14E-04	0.0394
TUSC3	8	15442130	29	−	3.73	6.7	4.28E-04	0.0496

Positive T-Values correspond with higher methylation in HPV(+) while negative T-Values correspond with higher methylation in HPV(−) tumors.

### CCNA1 Site Specific Validation

To validate our findings of increased *CCNA1* methylation HPV(+) tumors, we quantified methylation at 4 CpG sites in the promoter region of *CCNA1* in an additional 128 pretreatment head and neck tumors. Mean *CCNA1* methylation was significantly higher in HPV(+) tumors (p = 0.0005). After adjusting for age, sex, tumor site, and tumor stage, HPV(+) tumors were found to be, on average, 9.6% more methylated at the *CCNA1* promoter compared to HPV(−) tumors (p = 0.029).

### Gene Set Enrichment Analysis (GSEA)

To analyze if specific gene sets or pathways display differential epigenetic regulation in HPV(+) versus HPV(−) tumors, a GSEA of the genes associated with HPV status was conducted. An analysis of Gene Ontology (GO) Biological Processes significantly enriched for differentially methylated genes implies that three gene sets associated with cell cycle regulation were hypomethylated in HPV(+) tumors (**Table 3**). Specific genes included in these gene sets that were significantly less methylated in HPV(+) (p<0.05) include *RASSF1*, *CDK10*, *CHFR*, *RUNX3*, *APC*, and *CDKN2A* (*p16*). An analysis of enriched gene sets from the Kyoto Encyclopedia of Genes and Genomes (KEGG) found that genes associated with Neuroactive Ligand Receptor Interactions were hypermethylated in HPV(+) tumors (**Table 4**). The specific genes from this KEGG pathway include *GRPR*, *MC2R*, *GABRA5*, *PRSS1*, *NTSR1*, and *F2R.* Additionally, genes from the enriched KEGG set JAK-STAT Signaling Pathway were found to be hypomethylated in HPV(+) tumors, specifically *STAT5A*, *JAK3*, *OSM*, *MPL*, and *EPO*.

**Table 4:** Candidate enriched gene sets for differentially methylated genes associated with HPV status.

Name	Size	Enrichment Score (ES)	Normalized Enrichment Score (NES)	Nominal P-Value	FDR Q-Value
GENE ONTOLOGY - BIOLOGICAL PROCESSES					
Regulation of Cell Cycle	11	−0.54	−1.96	0.007	0.41
Cell Cycle (GO 0007049)	14	−0.43	−1.69	0.036	1
Negative Regulation of Cellular Metabolic Process	6	−0.58	−1.63	0.041	1
KEGG PATHWAYS					
Neuroactive Ligand Receptor Interaction (HSA04080)	6	0.6	1.72	0.02	0.27
JAK-STAT Signaling Pathway (HSA04630)	9	−0.49	−1.62	0.047	0.4

### CpG Sites Associated with Survival

Cox Proportional Hazards Regression was used to determine whether methylation at individual CpG sites is associated with three year survival rates. Significantly associated genes (p-value<0.05) are listed in **Table S2**. While no individual CpG site was found to have a false discovery rate less than 0.15, methylation at a number of genes was found to be potentially associated with survival, including *NOTCH1*, *UGT1A1*, and *IL-6.*

After comparing survival by cluster, where we noted that cases in Cluster 5 had significantly worse survival as well as apparent widespread differences in methylation, we conducted a post hoc analysis to identify specific genes differentially methylated in that cluster. After adjusting for other clinical covariates, including age, sex, cancer site, stage, smoking, problem drinking, and HPV status, a substantial number of genes were found to be differentially methylated in Cluster 5 compared to all other clusters. Gene set enrichment analysis identified pathways, molecular functions, and a chromosomal region significantly differentially methylated in Cluster 5 cases (**Table S3**). Genes located in chromosome 7q21 were found to be significantly hypomethylated in Cluster 5 cases. Biological processes associated with negative regulation of cellular metabolism as well as homeostatic processes were found to be enriched with genes hypomethylated in this cluster. An analysis of molecular functions identified dysregulation of nucleotide binding, particularly purine and adenyl nucleotide binding as well as kinase and phosphorus transferase activity.

## Discussion

Using an epidemiologically well characterized sample of head and neck cancer patients with a high proportion of HPV(+) cases, we confirmed a distinct epigenetic profile in HPV(+) head and neck cancers when compared to HPV(−) cancers. This has been previously noted by others for global methylation [15], candidate gene methylation [32] and by Marsit et al. using the same platform as this study [18]. Other studies have described the association between methylation and traditional risk factors for HPV(−) head and neck cancer such as smoking and alcohol use [33].

Our prior work has shown how epigenetic profiles and expression patterns correspond to these divergent mechanisms of carcinogenesis in HPV(+) and HPV(−) cell lines [19]. The findings of this study expand upon our prior cell line work, identifying numerous loci in tumor samples that are differentially methylated between HPV(+) and HPV(−) tumors, particularly those involved in cell cycle regulation and JAK-STAT signaling. The top differentially methylated site on the array between HPV(+) and HPV(−) tumors was seven bases downstream from the transcription start site of *CCNA1* with an average percent methylation level of 10% in HPV(−) tumors and 31% in HPV(+) tumors. This was also one of our top ranked genes in HNSCC cell lines [19], and was noted by other groups as differentially methylated [32] and differentially expressed [34] in HPV (+) HNSCC, indicating that methylation and expression of this gene could likely be important both mechanistically and as a biomarker for HPV-associated HNSCC. *CCNA1* is an important regulator of the cell cycle and is required for S phase and passage through G2 [35]. Other genes involved in cell cycle regulation tended to be hypomethylated in HPV(+) compared to HPV(−) HNSCC, indicating that regulation of these pathways may be important for HPV(+) head and neck carcinogenesis. This hypomethylated set of genes included many genes that have previously been shown to be methylated in head and neck cancer, including *RASSF1* [36], *CHFR* [37], *RUNX3* [38], *APC* [39], and *CDKN2A* (*p16*) [40]. These results are of particular importance to studies of biomarkers for head and neck cancer, which frequently do not take HPV status into account [41], [42], [43].

These analyses represent essentially a sizeable candidate-gene study, and the large number of loci allowed for initial pathway and positional analyses of the methylated CpGs. This was particularly useful when evaluating the contribution of epigenetic modifications to the prediction of survival, where methylation at single genes or sites did not predict survival time in this cohort. Hierarchical cluster analysis identified one set of patients with particularly worse survival solely based on methylation. Notably, this cluster did not include any HPV(+) cases, and contained the lowest proportion of males of all clusters (63%). This cluster had significant hypomethylation of 7q21, a region amplified in multiple cancers [44], [45]. This region has been identified as containing a placental-specific imprinted gene region [46], which is epigenetically inactivated in prostate carcinoma [47]. Thus, epigenetic regulation of this region may also play a role in a subset of head and neck cancers.

These and other epigenetic studies have strong implications for head and neck cancer research, particularly in light of recent reports on the complex landscape of head and neck cancer research [48], [49]. For example, the mutation rate of HPV-associated tumors was reported to be much lower than HPV(−) tumors by exome sequencing (from 2 to 5 times less likely to harbor mutations). The results of this study indicate that HPV-associated tumors are likely driven to a larger extent by methylation changes than HPV(−) tumors. Additionally, it is intriguing to hypothesize that methylation could serve as a complementary mechanism of inactivation in many known candidate tumor suppressor genes. For example, methylation of *NOTCH1* was the strongest predictor of survival in this study (p = 0.0002), and was also identified as frequently mutated in head and neck tumors in Stransky et al. and Agrawal et al. Interestingly, truncating mutations in *NOTCH1* indicate a tumor suppressor function as opposed to activating mutations seen in other cancers, and methylation of this gene also indicates a tumor suppressor function. Loss of heterozygosity (LOH) at the *NOTCH1* locus has also been reported for a small number of tumors [48]. The significance of the mechanism of inactivation remains to be clarified, but given the stable, yet potential reversible nature and variable levels of epigenetic modifications, this may have direct implications for treatment and therapy. Longitudinal epigenetic phenotyping of tumor methylation profiles during treatment could provide insight to the degree to which DNA methylation marks are labile to chemotherapy, radiation, or dietary intervention. These results also emphasize the importance of simultaneous evaluation of molecular mechanisms in tumors in conjunction with epidemiologic characteristics, and future studies will benefit from the careful existing comprehensive studies of molecular alterations in HNSCC.

This study has a number of limitations. The population size was relatively small, however, the technology used was able to detect differences in promoter methylation in a large number of genes associated with cancer. The Goldengate cancer panel, however, does not provide a measurement of promoter methylation in other genes with less well characterized functions, nor does it measure methylation at other genomic features, such as intergenic regions, which could provide information about genomic structure and stability. While the sample was representative of the patients seem in the institutions from which participants were recruited, women and particularly minorities were under-represented. Future planned studies will include a more diverse patient population and a more comprehensive view of the cancer epigenome, integrating epigenetic and transcriptional measures.

### Conclusions

Clinically and pathologically relevant subsets of tumors defined by methylation status have been identified in many cancer types, most notably the CpG Island Methylator Phenotype (CIMP) in colorectal cancer [50]. These CIMP tumors exhibit a distinct somatic profile of microsatellite instability and BRAF mutations, with divergent epidemiologic characteristics compared to non-CIMP tumors including a survival advantage [51], [52]. Array-based profiling of acute myeloid leukemias using the GoldenGate panel identified clinically relevant subgroups defined by epigenetic modifications, although there was not a strong association between these clusters and survival [53]. In this study we investigated the likelihood of identifying a clinically relevant subset of head and neck tumors defined by CpG methylation, taking advantage of a well-established patient cohort at the University of Michigan with well-annotated survival and epidemiologic data. Our sample was representative of the overall cohort regarding age, gender, smoking history, and alcohol consumption. We examined the epigenetic differences between HPV(+) and HPV(−) tumors, following from our recent work in cell lines showing evidence for divergent pathways of carcinogenesis and the well-described epidemiologic differences between individuals with differential HPV tumor status [19]. Further, we were able to evaluate survival in this cohort in light of their epigenetic profile (as defined by cluster status), HPV status and other epidemiologic characteristics.

## Supporting Information

Figure S1 Average differences in methylation per CpG site comparing HPV(+) and HPV(−) tumors.
(TIF)

Table S1 All CpG sites with DNA methylation values significantly associated (p<0.05) with HPV status of the tumor. Positive T-values correspond with sites more highly methylated in HPV(+) while negative T-values correspond with sites more highly methylated in HPV(−) tumors. Adjusted p-values were calculated via the Benjamini-Hochberg Method.
(DOCX)

Table S2 CpG sites identified as significantly associated (p<0.05) with three year survival by Cox Proportional Hazards Modeling.
(DOCX)

Table S3 Significantly enriched gene sets for genes identified as differentially methylated in cases from the cluster identified with worst survival (Cluster 5) compared to all other cases.
(DOCX)
