# Supplementary figures and images for: Comprehensive Analysis of DNA Methylation in Head and Neck Squamous Cell Carcinoma Indicates Differences by Survival and Clinicopathologic Characteristics
Source: PLoS One. 2013 Jan 24;8(1):e54742. doi: 10.1371/journal.pone.0054742 (PMC3554647; doi:10.1371/journal.pone.0054742)

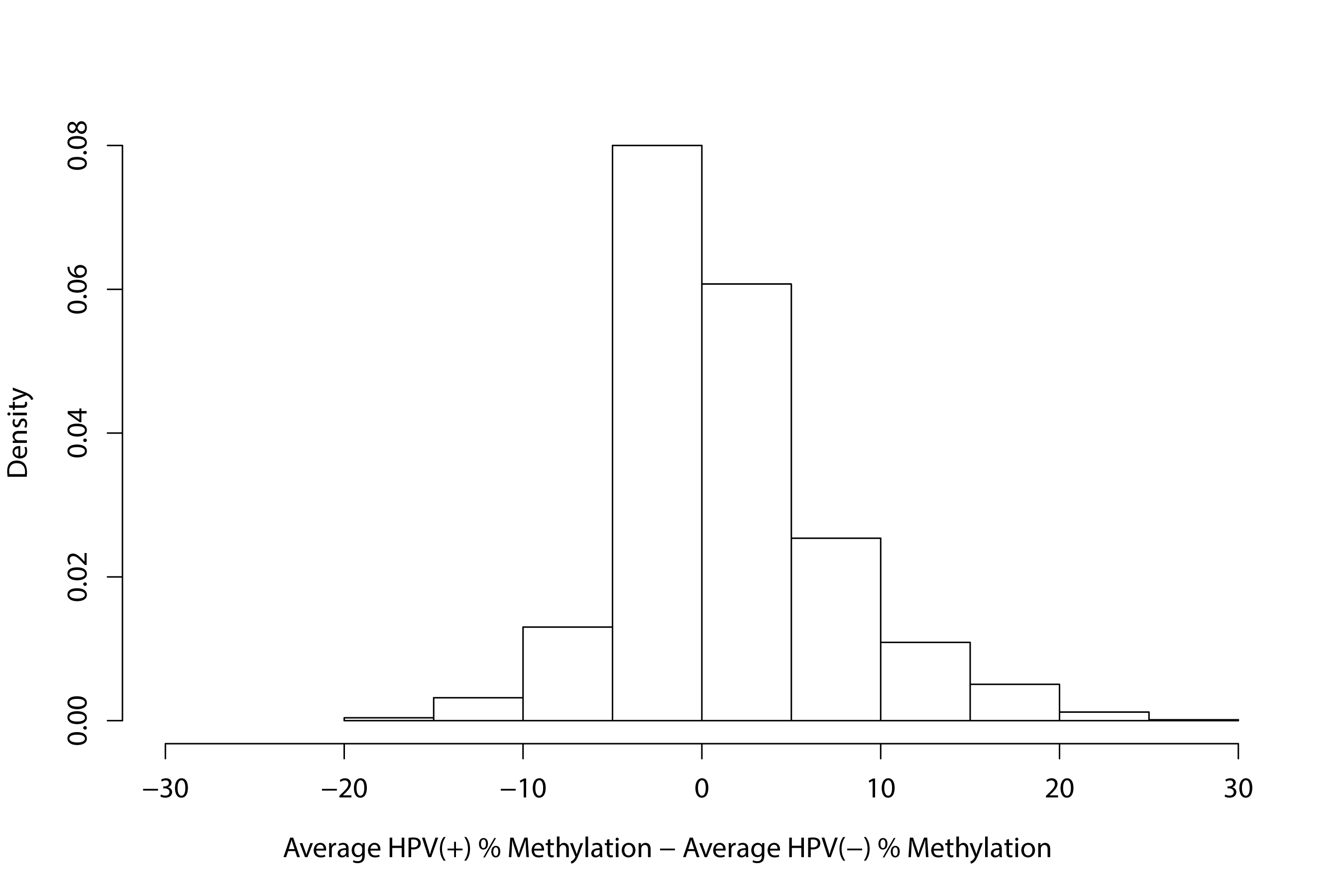

Supplement: Figure S1 — Average differences in methylation per CpG site comparing HPV(+) and HPV(−) tumors. (TIF) [file pone.0054742.s001.tif]
