# Supplementary material for: Comprehensive Analysis of DNA Methylation in Head and Neck Squamous Cell Carcinoma Indicates Differences by Survival and Clinicopathologic Characteristics
Source: PLoS One. 2013 Jan 24;8(1):e54742. doi: 10.1371/journal.pone.0054742 (PMC3554647; doi:10.1371/journal.pone.0054742)
Supplement: Table S1 — All CpG sites with DNA methylation values significantly associated (p<0.05) with HPV status of the tumor. Positive T-values correspond with sites more highly methylated in HPV(+) while negative T-values correspond with sites more highly methylated in HPV(−) tumors. Adjusted p-values were calculated via the Benjamini-Hochberg Method. (DOCX) [file pone.0054742.s002.docx]

| **Table S1**. All CpG sites with DNA methylation values significantly associated (p<0.05) with HPV status of the tumor. Positive T-values correspond with sites more highly methylated in HPV(+) while negative T-values correspond with sites more highly methylated in HPV(-) tumors. Adjusted p-values were calculated via the Benjamini-Hochberg Method. | | | | | | | |
| --- | --- | --- | --- | --- | --- | --- | --- |
| Gene Symbol | Chromosome | CpG Coordinate | Distance to TSS | DNA Strand of Transcription | T-Value | P-Value | Adjusted P-Value |
| CCNA1 | 13 | 35904640 | 7 | + | 5.30 | 1.86E-06 | 0.0028 |
| GRB7 | 17 | 35147553 | -160 | - | 4.58 | 2.46E-05 | 0.0161 |
| SPDEF | 6 | 34631953 | 116 | - | -4.51 | 3.20E-05 | 0.0161 |
| CDH11 | 16 | 63713774 | -354 | - | 4.32 | 6.08E-05 | 0.0192 |
| RUNX1T1 | 8 | 93176474 | 145 | - | 4.31 | 6.37E-05 | 0.0192 |
| RASSF1 | 3 | 50353615 | -244 | + | -4.22 | 8.47E-05 | 0.0213 |
| STAT5A | 17 | 37693133 | 42 | + | -4.05 | 1.51E-04 | 0.0318 |
| MGMT | 10 | 131155184 | -272 | - | -4.01 | 1.73E-04 | 0.0318 |
| ESR2 | 14 | 63830765 | 66 | + | -3.98 | 1.90E-04 | 0.0318 |
| JAK3 | 19 | 17819736 | 64 | + | -3.92 | 2.31E-04 | 0.0348 |
| SYBL1 | X | 154763858 | -349 | + | 3.88 | 2.71E-04 | 0.0370 |
| HSD17B12 | 11 | 43659026 | 145 | - | -3.83 | 3.14E-04 | 0.0394 |
| TUSC3 | 8 | 15442130 | 29 | - | 3.73 | 4.28E-04 | 0.0496 |
| TNFRSF10C | 8 | 23016372 | -7 | + | -3.66 | 5.38E-04 | 0.0578 |
| OSM | 22 | 28993028 | -188 | + | -3.61 | 6.39E-04 | 0.0629 |
| CD1A | 1 | 156490545 | -6 | + | 3.59 | 6.69E-04 | 0.0629 |
| PTPRG | 3 | 61522325 | 40 | - | -3.53 | 8.07E-04 | 0.0677 |
| CDK10 | 16 | 88280653 | 74 | + | -3.52 | 8.34E-04 | 0.0677 |
| NEFL | 8 | 24870155 | -209 | - | 3.52 | 8.55E-04 | 0.0677 |
| MPL | 1 | 43575405 | -657 | + | -3.44 | 0.0011 | 0.0806 |
| PLAT | 8 | 42184431 | -80 | + | 3.37 | 0.0013 | 0.0924 |
| TNFRSF10D | 8 | 23077555 | -70 | + | -3.37 | 0.0014 | 0.0924 |
| SEMA3F | 3 | 50168185 | 333 | - | 3.32 | 0.0016 | 0.0993 |
| IGF2AS | 11 | 2118120 | -203 | + | 3.31 | 0.0016 | 0.0993 |
| SMARCA4 | 19 | 10932244 | -362 | - | -3.28 | 0.0018 | 0.1064 |
| CHFR | 12 | 131974892 | -635 | - | -3.22 | 0.0021 | 0.1205 |
| ZNF264 | 19 | 62394284 | -397 | + | 3.21 | 0.0022 | 0.1206 |
| IRF7 | 11 | 605691 | 236 | - | -3.17 | 0.0024 | 0.1271 |
| MYLK | 3 | 125085707 | 132 | - | 3.17 | 0.0024 | 0.1271 |
| MEG3 | 14 | 100362306 | 91 | + | 3.12 | 0.0028 | 0.1381 |
| THBS2 | 6 | 169395933 | 129 | + | -3.12 | 0.0028 | 0.1381 |
| BCL2L2 | 14 | 22845586 | -280 | + | 3.10 | 0.0030 | 0.1388 |
| OSM | 22 | 28992874 | -34 | + | -3.06 | 0.0034 | 0.1491 |
| RASSF1 | 3 | 50353255 | 116 | + | -3.05 | 0.0034 | 0.1491 |
| SEMA3B | 3 | 50280140 | 96 | + | -3.03 | 0.0036 | 0.1491 |
| DES | 2 | 219991571 | 228 | - | -3.02 | 0.0037 | 0.1491 |
| GRPR | X | 16051145 | -200 | - | 3.01 | 0.0038 | 0.1491 |
| SHB | 9 | 38059683 | -473 | - | -3.01 | 0.0038 | 0.1491 |
| SPI1 | 11 | 47356469 | 205 | + | -3.01 | 0.0039 | 0.1491 |
| IRF5 | 7 | 128365107 | -123 | + | -2.97 | 0.0043 | 0.1609 |
| CREBBP | 16 | 3871424 | -712 | - | 2.96 | 0.0044 | 0.1614 |
| HPSE | 4 | 84475422 | -93 | + | -2.95 | 0.0045 | 0.1624 |
| ICAM1 | 19 | 10242393 | -386 | - | -2.94 | 0.0046 | 0.1624 |
| EYA4 | 6 | 133603698 | -508 | + | -2.86 | 0.0059 | 0.1984 |
| ADAMTS12 | 5 | 33927829 | 52 | - | -2.85 | 0.0060 | 0.1984 |
| PTPRO | 12 | 15366383 | -371 | + | -2.84 | 0.0061 | 0.1984 |
| S100A2 | 1 | 151804894 | 36 | - | 2.84 | 0.0063 | 0.1984 |
| CASP8 | 2 | 201806900 | 474 | + | -2.82 | 0.0065 | 0.1984 |
| CTGF | 6 | 132314848 | -693 | - | 2.82 | 0.0066 | 0.1984 |
| E2F5 | 8 | 86276358 | -516 | - | -2.82 | 0.0066 | 0.1984 |
| CTSL | 9 | 89530719 | -81 | + | 2.80 | 0.0069 | 0.2002 |
| NDN | 15 | 21483412 | 131 | - | 2.80 | 0.0069 | 0.2002 |
| SHH | 7 | 155297400 | 328 | + | 2.79 | 0.0071 | 0.2021 |
| RIPK4 | 21 | 42060152 | 166 | + | 2.78 | 0.0073 | 0.2042 |
| DSC2 | 18 | 26936285 | 90 | + | 2.76 | 0.0077 | 0.2073 |
| FRK | 6 | 116488650 | -36 | + | 2.76 | 0.0077 | 0.2073 |
| CCNA1 | 13 | 35904417 | -216 | + | 2.75 | 0.0080 | 0.2107 |
| CSF3R | 1 | 36721104 | -8 | + | 2.72 | 0.0086 | 0.2141 |
| CHFR | 12 | 131974758 | -501 | + | -2.71 | 0.0087 | 0.2141 |
| EPHA7 | 6 | 94186198 | -205 | - | -2.71 | 0.0087 | 0.2141 |
| RUNX3 | 1 | 25164455 | -393 | - | -2.71 | 0.0088 | 0.2141 |
| AXL | 19 | 46416440 | -223 | - | 2.71 | 0.0089 | 0.2141 |
| HLA-DOB | 6 | 32893119 | -357 | - | -2.70 | 0.0091 | 0.2141 |
| FGF1 | 5 | 142046169 | -357 | - | 2.70 | 0.0092 | 0.2141 |
| NOTCH4 | 6 | 32299818 | 4 | + | 2.69 | 0.0093 | 0.2141 |
| RAB32 | 6 | 146906835 | 314 | - | 2.69 | 0.0094 | 0.2141 |
| SMO | 7 | 128616006 | 57 | + | -2.68 | 0.0096 | 0.2151 |
| CDKN2A | 9 | 21964709 | 229 | - | -2.67 | 0.0098 | 0.2164 |
| MC2R | 18 | 13906560 | -1025 | + | 2.66 | 0.0101 | 0.2193 |
| MMP9 | 20 | 44070765 | -189 | + | 2.62 | 0.0111 | 0.2354 |
| GABRA5 | 15 | 24742740 | 44 | - | 2.62 | 0.0112 | 0.2354 |
| SPI1 | 11 | 47357603 | -929 | + | 2.62 | 0.0113 | 0.2354 |
| HSPA2 | 14 | 64072214 | -162 | - | -2.60 | 0.0117 | 0.2405 |
| SOX2 | 3 | 182911870 | -546 | + | -2.59 | 0.0121 | 0.2405 |
| GADD45A | 1 | 67922734 | -737 | - | 2.58 | 0.0124 | 0.2405 |
| TNK1 | 17 | 7225093 | -41 | - | 2.58 | 0.0124 | 0.2405 |
| JAK3 | 19 | 17819956 | -156 | - | -2.58 | 0.0124 | 0.2405 |
| CDH11 | 16 | 63713623 | -203 | - | 2.58 | 0.0125 | 0.2405 |
| FASTK | 7 | 150409141 | -257 | + | 2.57 | 0.0128 | 0.2405 |
| MMP14 | 14 | 22375425 | -208 | - | 2.57 | 0.0128 | 0.2405 |
| EPHB4 | 7 | 100263392 | -313 | - | -2.55 | 0.0134 | 0.2487 |
| RHOH | 4 | 39874876 | -121 | + | -2.54 | 0.0139 | 0.2556 |
| COL18A1 | 21 | 45649031 | -494 | - | -2.51 | 0.0147 | 0.2619 |
| IGFBP5 | 2 | 217268372 | 144 | + | -2.51 | 0.0149 | 0.2619 |
| FGF7 | 15 | 47502707 | -44 | + | 2.51 | 0.0150 | 0.2619 |
| PRSS1 | 7 | 142136949 | 45 | - | 2.51 | 0.0150 | 0.2619 |
| CSF2 | 5 | 131437632 | 248 | - | 2.50 | 0.0151 | 0.2619 |
| SMO | 7 | 128615494 | -455 | - | 2.50 | 0.0153 | 0.2619 |
| HDAC6 | X | 48545533 | 102 | + | 2.49 | 0.0155 | 0.2629 |
| SERPINE1 | 7 | 100556653 | -519 | + | 2.49 | 0.0158 | 0.2641 |
| TGFA | 2 | 70634996 | -558 | + | -2.48 | 0.0161 | 0.2661 |
| HRASLS | 3 | 194441259 | -353 | - | 2.47 | 0.0164 | 0.2669 |
| TRIM29 | 11 | 119514334 | -261 | + | 2.47 | 0.0165 | 0.2669 |
| SLIT2 | 4 | 19864125 | -208 | + | 2.46 | 0.0167 | 0.2669 |
| CD40 | 20 | 44179941 | -372 | - | -2.45 | 0.0172 | 0.2700 |
| GNMT | 6 | 43036604 | 126 | + | -2.45 | 0.0172 | 0.2700 |
| HOXA11 | 7 | 27191320 | 35 | + | -2.44 | 0.0178 | 0.2726 |
| PLA2G2A | 1 | 20179228 | 268 | + | -2.43 | 0.0182 | 0.2726 |
| PTPNS1 | 20 | 1823858 | 433 | - | -2.43 | 0.0182 | 0.2726 |
| NOTCH1 | 9 | 138559607 | 452 | - | -2.43 | 0.0182 | 0.2726 |
| YES1 | 18 | 802927 | -600 | + | -2.43 | 0.0183 | 0.2726 |
| JAK3 | 19 | 17820875 | -1075 | - | 2.42 | 0.0185 | 0.2727 |
| FLT3 | 13 | 27573007 | -302 | + | -2.42 | 0.0188 | 0.2745 |
| MET | 7 | 116100028 | 333 | + | 2.41 | 0.0191 | 0.2757 |
| CD9 | 12 | 6179231 | -585 | - | 2.40 | 0.0194 | 0.2785 |
| GDF10 | 10 | 48059133 | 39 | + | -2.39 | 0.0200 | 0.2827 |
| MATK | 19 | 3752874 | -64 | + | -2.39 | 0.0201 | 0.2827 |
| FGFR2 | 10 | 123348367 | -460 | - | 2.38 | 0.0207 | 0.2886 |
| KCNK4 | 11 | 63815280 | -171 | - | -2.37 | 0.0209 | 0.2886 |
| RHOH | 4 | 39874044 | -953 | - | 2.35 | 0.0222 | 0.3036 |
| INS | 11 | 2139248 | -248 | + | 2.34 | 0.0229 | 0.3089 |
| PLS3 | X | 114701835 | 70 | + | 2.33 | 0.0230 | 0.3089 |
| APC | 5 | 112101600 | 117 | - | -2.33 | 0.0232 | 0.3095 |
| APC | 5 | 112101203 | -280 | - | -2.32 | 0.0237 | 0.3124 |
| ZNF264 | 19 | 62394729 | 48 | - | -2.32 | 0.0240 | 0.3144 |
| GUCY2D | 17 | 7846665 | -48 | - | -2.31 | 0.0243 | 0.3151 |
| CD40 | 20 | 44180371 | 58 | - | -2.31 | 0.0247 | 0.3170 |
| EPHA2 | 1 | 16355354 | -203 | + | 2.30 | 0.0251 | 0.3170 |
| ETV1 | 7 | 13995804 | -515 | + | -2.30 | 0.0251 | 0.3170 |
| MAPK10 | 4 | 87593281 | 26 | + | 2.30 | 0.0253 | 0.3170 |
| ACVR1 | 2 | 158404019 | -983 | + | 2.29 | 0.0259 | 0.3221 |
| AATK | 17 | 76710603 | -709 | - | 2.27 | 0.0269 | 0.3318 |
| IMPACT | 18 | 20260446 | -234 | - | 2.26 | 0.0273 | 0.3319 |
| LOX | 5 | 121442166 | -313 | - | 2.26 | 0.0274 | 0.3319 |
| MGMT | 10 | 131155175 | -281 | + | -2.26 | 0.0276 | 0.3319 |
| BTK | X | 100527943 | -105 | + | -2.25 | 0.0281 | 0.3361 |
| NTSR1 | 20 | 60810316 | -318 | + | 2.25 | 0.0284 | 0.3361 |
| ASCL2 | 11 | 2249367 | -609 | - | 2.24 | 0.0291 | 0.3424 |
| MKRN4 | X | 40578589 | 249 | - | 2.22 | 0.0306 | 0.3453 |
| ICAM1 | 19 | 10243021 | 242 | + | -2.21 | 0.0309 | 0.3453 |
| IGSF4C | 19 | 48836364 | -533 | - | -2.21 | 0.0311 | 0.3453 |
| CD9 | 12 | 6179312 | -504 | + | 2.21 | 0.0312 | 0.3453 |
| TIE1 | 1 | 43539317 | 66 | - | 2.21 | 0.0312 | 0.3453 |
| RUNX3 | 1 | 25164035 | 27 | - | -2.20 | 0.0315 | 0.3453 |
| SPDEF | 6 | 34632075 | -6 | - | 2.20 | 0.0315 | 0.3453 |
| ETV6 | 12 | 11694485 | 430 | + | -2.20 | 0.0319 | 0.3453 |
| CD86 | 3 | 123256908 | -3 | + | 2.20 | 0.0319 | 0.3453 |
| PAX6 | 11 | 31790576 | -1121 | + | -2.20 | 0.0319 | 0.3453 |
| INHA | 2 | 220144009 | 1252 | + | -2.20 | 0.0321 | 0.3453 |
| EPO | 7 | 100156603 | 244 | - | -2.19 | 0.0323 | 0.3453 |
| DBC1 | 9 | 121171318 | 204 | + | 2.19 | 0.0325 | 0.3453 |
| FLT3 | 13 | 27572379 | 326 | - | -2.19 | 0.0326 | 0.3453 |
| KRT5 | 12 | 51200818 | -308 | + | 2.19 | 0.0329 | 0.3458 |
| IL4 | 5 | 132037010 | -262 | - | 2.17 | 0.0344 | 0.3592 |
| SLIT2 | 4 | 19864444 | 111 | - | -2.16 | 0.0353 | 0.3660 |
| MMP10 | 11 | 102156418 | 136 | - | 2.15 | 0.0357 | 0.3680 |
| CDKN1C | 11 | 2864177 | -626 | + | -2.14 | 0.0362 | 0.3706 |
| UBA52 | 19 | 18543375 | -293 | - | -2.14 | 0.0366 | 0.3706 |
| MMP14 | 14 | 22375620 | -13 | + | 2.14 | 0.0367 | 0.3706 |
| EMR3 | 19 | 14646849 | -39 | - | 2.13 | 0.0375 | 0.3739 |
| GSTM1 | 1 | 110031699 | -266 | + | 2.13 | 0.0375 | 0.3739 |
| DSC2 | 18 | 26936782 | -407 | - | 2.12 | 0.0378 | 0.3739 |
| ARHGAP9 | 12 | 56169124 | -260 | + | 2.12 | 0.0383 | 0.3739 |
| COL1A2 | 7 | 93861402 | -407 | - | 2.12 | 0.0383 | 0.3739 |
| APOC2 | 19 | 50140706 | -377 | + | 2.11 | 0.0389 | 0.3746 |
| STK11 | 19 | 1156503 | -295 | - | -2.11 | 0.0392 | 0.3746 |
| KIAA1804 | 1 | 231529448 | -689 | - | 2.11 | 0.0392 | 0.3746 |
| F2R | 5 | 76047454 | -88 | + | 2.11 | 0.0393 | 0.3746 |
| IGSF4 | 11 | 114880779 | -454 | + | 2.10 | 0.0398 | 0.3763 |
| CDH11 | 16 | 63713318 | 102 | - | 2.10 | 0.0400 | 0.3763 |
| RARB | 3 | 25444698 | -60 | + | 2.09 | 0.0410 | 0.3790 |
| HIC2 | 22 | 20101165 | -528 | - | 2.09 | 0.0411 | 0.3790 |
| SEMA3A | 7 | 83662191 | -343 | + | -2.09 | 0.0414 | 0.3790 |
| ITK | 5 | 156540651 | 166 | - | -2.08 | 0.0416 | 0.3790 |
| NRAS | 1 | 115061141 | -103 | - | 2.08 | 0.0421 | 0.3790 |
| PTGS2 | 1 | 184916703 | -524 | - | 2.07 | 0.0425 | 0.3790 |
| RIPK2 | 8 | 90839296 | 123 | + | -2.07 | 0.0425 | 0.3790 |
| RUNX3 | 1 | 25164309 | -247 | + | -2.07 | 0.0426 | 0.3790 |
| COL1A1 | 17 | 45633997 | -5 | + | 2.07 | 0.0432 | 0.3790 |
| TCF4 | 18 | 51406615 | -175 | - | -2.07 | 0.0432 | 0.3790 |
| SMAD4 | 18 | 46810137 | -474 | - | -2.07 | 0.0432 | 0.3790 |
| THY1 | 11 | 118799110 | -20 | - | -2.06 | 0.0434 | 0.3790 |
| COL18A1 | 21 | 45649160 | -365 | - | 2.06 | 0.0436 | 0.3790 |
| FES | 15 | 89228490 | -223 | - | -2.06 | 0.0439 | 0.3799 |
| GNAS | 20 | 56848248 | 58 | + | 2.05 | 0.0451 | 0.3873 |
| PTHLH | 12 | 28016940 | -757 | + | 2.05 | 0.0453 | 0.3873 |
| PITX2 | 4 | 111777933 | 24 | - | 2.04 | 0.0457 | 0.3887 |
| MCM6 | 2 | 136350345 | 136 | + | -2.04 | 0.0461 | 0.3896 |
| EPHA8 | 1 | 22762135 | -456 | - | -2.03 | 0.0470 | 0.3939 |
| MYCN | 2 | 15998211 | 77 | - | -2.03 | 0.0471 | 0.3939 |
| MYOD1 | 11 | 17697891 | 156 | + | 2.02 | 0.0476 | 0.3960 |
| RYK | 3 | 135452769 | -493 | + | -2.02 | 0.0481 | 0.3974 |
| RRAS | 19 | 54835312 | -100 | - | -2.01 | 0.0487 | 0.4004 |
| SEPT9 | 17 | 72827370 | -374 | + | 2.01 | 0.0490 | 0.4005 |
| MME | 3 | 156280182 | 29 | + | 2.00 | 0.0496 | 0.4005 |
| GPR116 | 6 | 46991675 | -850 | + | 2.00 | 0.0497 | 0.4005 |
| DSG1 | 18 | 27151891 | -159 | - | 2.00 | 0.0498 | 0.4005 |
