# Supplementary material for: Comprehensive Analysis of DNA Methylation in Head and Neck Squamous Cell Carcinoma Indicates Differences by Survival and Clinicopathologic Characteristics
Source: PLoS One. 2013 Jan 24;8(1):e54742. doi: 10.1371/journal.pone.0054742 (PMC3554647; doi:10.1371/journal.pone.0054742)
Supplement: Table S2 — CpG sites identified as significantly associated (p<0.05) with three year survival by Cox Proportional Hazards Modeling. (DOCX) [file pone.0054742.s003.docx]

| Table S2. CpG sites identified as significantly associated (p<0.05) with three year survival by Cox Proportional Hazards Modeling. | | | | | | | | |
| --- | --- | --- | --- | --- | --- | --- | --- | --- |
| Gene Symbol | Chromosome | CpG Coordinate | Distance to TSS | DNA Strand of Transcription | Hazard Coefficient | Standard Error | P-Value | Q-Value |
| NOTCH1 | 9 | 138559607 | 452 | - | 22.29 | 5.95 | 0.0002 | 0.172 |
| TEK | 9 | 27098915 | -526 | + | -5.66 | 1.63 | 0.0005 | 0.172 |
| UGT1A1 | 2 | 234333094 | -564 | - | -6.63 | 1.92 | 0.0005 | 0.172 |
| IL6 | 7 | 22732734 | -611 | + | 4.86 | 1.45 | 0.0008 | 0.172 |
| AHR | 7 | 17304605 | -166 | - | 12.36 | 3.82 | 0.0012 | 0.172 |
| RRAS | 19 | 54835312 | -100 | - | 11.86 | 3.69 | 0.0013 | 0.172 |
| NEO1 | 15 | 71130861 | -1067 | + | 22.72 | 7.17 | 0.0015 | 0.172 |
| F2R | 5 | 76046703 | -839 | + | -4.45 | 1.41 | 0.0016 | 0.172 |
| IGFBP3 | 7 | 45928431 | -1035 | + | 11.24 | 3.58 | 0.0017 | 0.172 |
| PLS3 | X | 114701671 | -94 | - | 8.04 | 2.58 | 0.0019 | 0.172 |
| HS3ST2 | 16 | 22732815 | -546 | + | -6.21 | 2.00 | 0.0019 | 0.172 |
| MST1R | 3 | 49916466 | -392 | + | 13.09 | 4.22 | 0.0019 | 0.172 |
| MYOD1 | 11 | 17697891 | 156 | + | -4.41 | 1.44 | 0.0021 | 0.175 |
| FER | 5 | 108110841 | -581 | + | -4.86 | 1.64 | 0.0030 | 0.215 |
| PLAT | 8 | 42184431 | -80 | + | 12.04 | 4.06 | 0.0030 | 0.215 |
| VAMP8 | 2 | 85657987 | -241 | + | 6.68 | 2.29 | 0.0035 | 0.217 |
| ARHGAP9 | 12 | 56169124 | -260 | + | -3.76 | 1.31 | 0.0039 | 0.217 |
| TMEFF1 | 9 | 102275718 | 180 | - | 7.56 | 2.63 | 0.0041 | 0.217 |
| CYP1B1 | 2 | 38157008 | -212 | + | 4.89 | 1.71 | 0.0042 | 0.217 |
| JAG2 | 14 | 104706470 | -264 | + | 15.37 | 5.38 | 0.0043 | 0.217 |
| TMEFF2 | 2 | 192767395 | 494 | - | -3.70 | 1.30 | 0.0043 | 0.217 |
| ROR1 | 1 | 64012296 | -6 | + | 4.60 | 1.64 | 0.0049 | 0.240 |
| RUNX3 | 1 | 25164035 | 27 | - | -3.52 | 1.26 | 0.0053 | 0.246 |
| EVI2A | 17 | 26672423 | 420 | + | -3.78 | 1.37 | 0.0058 | 0.246 |
| S100A4 | 1 | 151785100 | -194 | - | -3.35 | 1.22 | 0.0059 | 0.246 |
| BTK | X | 100527943 | -105 | + | -6.03 | 2.19 | 0.0060 | 0.246 |
| CDC25B | 20 | 3724375 | -11 | - | 28.47 | 10.46 | 0.0065 | 0.258 |
| DSP | 6 | 7486833 | -36 | + | 9.05 | 3.35 | 0.0069 | 0.262 |
| CFTR | 7 | 116906881 | -372 | - | -4.54 | 1.70 | 0.0075 | 0.263 |
| NTSR1 | 20 | 60810743 | 109 | + | 6.76 | 2.54 | 0.0077 | 0.263 |
| MC2R | 18 | 13906560 | -1025 | + | 3.51 | 1.32 | 0.0080 | 0.263 |
| JAK2 | 9 | 4974473 | -772 | - | 8.76 | 3.30 | 0.0080 | 0.263 |
| HTR1B | 6 | 78229607 | 232 | - | -3.58 | 1.35 | 0.0081 | 0.263 |
| RIPK4 | 21 | 42060490 | -172 | + | 4.01 | 1.53 | 0.0087 | 0.275 |
| IRAK1 | X | 152938848 | -312 | + | 5.69 | 2.18 | 0.0091 | 0.278 |
| JAK3 | 19 | 17820875 | -1075 | - | -3.22 | 1.24 | 0.0093 | 0.278 |
| BCL2A1 | 15 | 78051825 | -1127 | - | -3.05 | 1.18 | 0.0098 | 0.279 |
| CPA4 | 7 | 129719269 | -961 | - | -2.81 | 1.10 | 0.0105 | 0.279 |
| FANCA | 16 | 88411572 | -1006 | - | -2.97 | 1.16 | 0.0105 | 0.279 |
| LAMC1 | 1 | 181259642 | 466 | - | 35.82 | 14.07 | 0.0109 | 0.279 |
| PTK6 | 20 | 61639101 | 50 | + | 3.65 | 1.44 | 0.0111 | 0.279 |
| YES1 | 18 | 802927 | -600 | + | 22.56 | 8.92 | 0.0114 | 0.279 |
| VAV2 | 9 | 135847168 | 58 | + | 31.29 | 12.40 | 0.0116 | 0.279 |
| JUNB | 19 | 12762161 | -1149 | - | 7.59 | 3.01 | 0.0118 | 0.279 |
| PCDH1 | 5 | 141238392 | -264 | + | 7.53 | 3.01 | 0.0124 | 0.279 |
| SEMA3F | 3 | 50168185 | 333 | - | 12.85 | 5.14 | 0.0125 | 0.279 |
| ZIM3 | 19 | 62348179 | 203 | + | -4.55 | 1.82 | 0.0125 | 0.279 |
| SNURF | 15 | 22751226 | -2 | - | -3.91 | 1.57 | 0.0127 | 0.279 |
| FES | 15 | 89228490 | -223 | - | -5.26 | 2.11 | 0.0129 | 0.279 |
| LYN | 8 | 56955279 | 353 | + | 5.58 | 2.25 | 0.0131 | 0.279 |
| NPR2 | 9 | 35781788 | -618 | + | 3.91 | 1.58 | 0.0133 | 0.279 |
| MLH1 | 3 | 37009602 | 197 | + | 20.70 | 8.43 | 0.0140 | 0.284 |
| ICAM1 | 19 | 10242393 | -386 | - | 6.95 | 2.84 | 0.0142 | 0.284 |
| ALPL | 1 | 21708178 | -278 | + | 15.42 | 6.30 | 0.0143 | 0.284 |
| ITGA2 | 5 | 52321134 | 120 | + | 16.56 | 6.81 | 0.0151 | 0.284 |
| RBP1 | 3 | 140741330 | -150 | + | 10.78 | 4.45 | 0.0153 | 0.284 |
| EPHA8 | 1 | 22762135 | -456 | - | -4.16 | 1.71 | 0.0153 | 0.284 |
| ATP10A | 15 | 23660487 | -524 | - | -3.81 | 1.57 | 0.0155 | 0.284 |
| TDGF1 | 3 | 46593789 | -428 | - | -3.13 | 1.30 | 0.0161 | 0.284 |
| MYH11 | 16 | 15858391 | -22 | + | -4.50 | 1.87 | 0.0162 | 0.284 |
| MFAP4 | 17 | 19231283 | -197 | + | -3.45 | 1.44 | 0.0163 | 0.284 |
| MMP19 | 12 | 54522728 | 274 | - | -2.74 | 1.14 | 0.0167 | 0.284 |
| MAP3K1 | 5 | 56146015 | -7 | + | 12.84 | 5.37 | 0.0168 | 0.284 |
| PRKCDBP | 11 | 6298110 | 206 | + | 5.20 | 2.18 | 0.0170 | 0.284 |
| MFAP4 | 17 | 19231096 | -10 | - | -3.96 | 1.67 | 0.0177 | 0.289 |
| SH3BP2 | 4 | 2789568 | -771 | - | 10.49 | 4.44 | 0.0182 | 0.289 |
| LRP2 | 2 | 169927239 | 20 | + | 2.32 | 0.98 | 0.0184 | 0.289 |
| PHLDA2 | 11 | 2907848 | -622 | + | 6.22 | 2.64 | 0.0184 | 0.289 |
| SEMA3A | 7 | 83662506 | -658 | - | -8.23 | 3.50 | 0.0187 | 0.289 |
| PLS3 | X | 114701835 | 70 | + | 2.54 | 1.08 | 0.0189 | 0.289 |
| DDR2 | 1 | 160869183 | 331 | + | -2.97 | 1.27 | 0.0192 | 0.289 |
| SMAD2 | 18 | 43712069 | -848 | - | 12.81 | 5.50 | 0.0199 | 0.289 |
| PGF | 14 | 74492011 | 33 | + | 8.42 | 3.62 | 0.0201 | 0.289 |
| INHA | 2 | 220144009 | 1252 | + | 9.52 | 4.10 | 0.0203 | 0.289 |
| BCL3 | 19 | 49943942 | 71 | + | 4.88 | 2.11 | 0.0205 | 0.289 |
| CTLA4 | 2 | 204440932 | 176 | - | -2.54 | 1.10 | 0.0205 | 0.289 |
| IL3 | 5 | 131423690 | -556 | + | -2.18 | 0.94 | 0.0208 | 0.290 |
| GAS7 | 17 | 10042445 | 148 | + | -2.54 | 1.10 | 0.0213 | 0.293 |
| MYCN | 2 | 15997670 | -464 | - | 16.94 | 7.47 | 0.0233 | 0.316 |
| EPHX1 | 1 | 224079751 | 152 | + | -2.69 | 1.19 | 0.0236 | 0.316 |
| SOD3 | 4 | 24404928 | -225 | + | -2.31 | 1.03 | 0.0244 | 0.321 |
| SLC5A8 | 12 | 100128060 | 60 | - | -3.05 | 1.36 | 0.0248 | 0.321 |
| EGR4 | 2 | 73374048 | 70 | + | 3.77 | 1.68 | 0.0251 | 0.321 |
| LMO1 | 11 | 8241717 | 265 | - | -23.80 | 10.66 | 0.0255 | 0.321 |
| BLK | 8 | 11388916 | -14 | + | -3.08 | 1.38 | 0.0258 | 0.321 |
| MUSK | 9 | 112470652 | -308 | + | -2.44 | 1.10 | 0.0261 | 0.321 |
| EYA4 | 6 | 133603412 | -794 | + | -2.50 | 1.13 | 0.0266 | 0.321 |
| ZNF264 | 19 | 62394729 | 48 | - | 7.10 | 3.20 | 0.0267 | 0.321 |
| PPARG | 3 | 12304537 | 178 | - | 4.19 | 1.89 | 0.0268 | 0.321 |
| EDN1 | 6 | 12398606 | -39 | - | 20.69 | 9.35 | 0.0269 | 0.321 |
| BMPR2 | 2 | 202950351 | 435 | + | 41.33 | 18.84 | 0.0283 | 0.330 |
| MYLK | 3 | 125086308 | -469 | - | -5.43 | 2.48 | 0.0286 | 0.330 |
| GLI3 | 7 | 42241564 | 148 | - | -3.45 | 1.58 | 0.0286 | 0.330 |
| PWCR1 | 15 | 22846906 | -811 | + | -2.38 | 1.09 | 0.0294 | 0.335 |
| MAF | 16 | 78192938 | -826 | - | 15.89 | 7.31 | 0.0297 | 0.335 |
| MYBL2 | 20 | 41728769 | -354 | + | 9.32 | 4.31 | 0.0307 | 0.343 |
| EMR3 | 19 | 14646849 | -39 | - | 5.10 | 2.37 | 0.0310 | 0.343 |
| HLA-DPA1 | 6 | 33149321 | 35 | - | -2.52 | 1.18 | 0.0323 | 0.353 |
| TNK1 | 17 | 7224913 | -221 | + | -2.98 | 1.40 | 0.0327 | 0.354 |
| IL8 | 4 | 74825056 | -83 | + | -4.95 | 2.32 | 0.0331 | 0.354 |
| XRCC1 | 19 | 48772236 | -681 | - | -2.12 | 1.00 | 0.0342 | 0.362 |
| KIAA1804 | 1 | 231529448 | -689 | - | 3.08 | 1.46 | 0.0345 | 0.362 |
| PROK2 | 3 | 71916902 | 0 | + | 2.16 | 1.02 | 0.0349 | 0.363 |
| GPX1 | 3 | 49370989 | -194 | + | 9.52 | 4.53 | 0.0356 | 0.364 |
| EVI1 | 3 | 170346817 | -30 | - | -19.54 | 9.32 | 0.0361 | 0.364 |
| KRAS | 12 | 25295039 | 82 | + | -3.42 | 1.63 | 0.0361 | 0.364 |
| ACVR1 | 2 | 158402708 | 328 | - | -2.23 | 1.07 | 0.0363 | 0.364 |
| PLG | 6 | 161043679 | 406 | + | -2.26 | 1.08 | 0.0370 | 0.366 |
| C4B | 6 | 32057622 | -191 | + | -2.72 | 1.30 | 0.0373 | 0.366 |
| ITGB4 | 17 | 71228594 | -517 | + | -6.47 | 3.11 | 0.0375 | 0.366 |
| LMO1 | 11 | 8242151 | -169 | + | 17.98 | 8.67 | 0.0380 | 0.367 |
| S100A4 | 1 | 151785793 | -887 | - | -2.55 | 1.24 | 0.0389 | 0.372 |
| AXL | 19 | 46416440 | -223 | - | -4.67 | 2.27 | 0.0393 | 0.372 |
| TDGF1 | 3 | 46594270 | 53 | - | -1.97 | 0.96 | 0.0410 | 0.376 |
| SEPT9 | 17 | 72827686 | -58 | - | -1.77 | 0.87 | 0.0413 | 0.376 |
| RASA1 | 5 | 86600014 | 107 | + | 13.88 | 6.80 | 0.0413 | 0.376 |
| SOX1 | 13 | 111768896 | -1018 | - | -6.54 | 3.21 | 0.0414 | 0.376 |
| GSTP1 | 11 | 67107788 | -74 | + | -25.67 | 12.59 | 0.0415 | 0.376 |
| LTA | 6 | 31647858 | -214 | - | -2.09 | 1.03 | 0.0419 | 0.376 |
| BDNF | 11 | 27700131 | -259 | - | -2.10 | 1.03 | 0.0423 | 0.376 |
| SMAD2 | 18 | 43711929 | -708 | - | -76.61 | 37.81 | 0.0427 | 0.376 |
| CD34 | 1 | 206152086 | -780 | - | -2.56 | 1.27 | 0.0435 | 0.376 |
| RYK | 3 | 135452769 | -493 | + | 6.38 | 3.16 | 0.0435 | 0.376 |
| FVT1 | 18 | 59185663 | -225 | + | 2.17 | 1.07 | 0.0436 | 0.376 |
| CD81 | 11 | 2354912 | -211 | + | -2.66 | 1.32 | 0.0444 | 0.380 |
| RET | 10 | 42892544 | 11 | + | -3.52 | 1.76 | 0.0451 | 0.380 |
| TSC2 | 16 | 2038740 | 140 | + | -8.57 | 4.28 | 0.0452 | 0.380 |
| SLC6A8 | X | 152606393 | -193 | - | 2.76 | 1.38 | 0.0460 | 0.380 |
| MME | 3 | 156279765 | -388 | + | -3.47 | 1.74 | 0.0462 | 0.380 |
| HHIP | 4 | 145786045 | -578 | - | -5.15 | 2.58 | 0.0463 | 0.380 |
| CCKAR | 4 | 26101061 | 79 | + | -1.94 | 0.98 | 0.0467 | 0.380 |
| CDK6 | 7 | 92300892 | 256 | + | 23.63 | 11.90 | 0.0471 | 0.380 |
| HOXA11 | 7 | 27191447 | -92 | - | 3.00 | 1.52 | 0.0479 | 0.380 |
| TMEFF1 | 9 | 102274912 | -626 | - | -4.52 | 2.29 | 0.0483 | 0.380 |
| TGFBR3 | 1 | 92124672 | -429 | + | 7.68 | 3.89 | 0.0486 | 0.380 |
