# Supplementary material for: Comprehensive Analysis of DNA Methylation in Head and Neck Squamous Cell Carcinoma Indicates Differences by Survival and Clinicopathologic Characteristics
Source: PLoS One. 2013 Jan 24;8(1):e54742. doi: 10.1371/journal.pone.0054742 (PMC3554647; doi:10.1371/journal.pone.0054742)
Supplement: Table S3 — Significantly enriched gene sets for genes identified as differentially methylated in cases from the cluster identified with worst survival (Cluster 5) compared to all other cases. (DOCX) [file pone.0054742.s004.docx]

| Table S3. Significantly enriched gene sets for genes identified as differentially methylated in cases from the cluster identified with worst survival (Cluster 5) compared to all other cases. | | | | | |
| --- | --- | --- | --- | --- | --- |
| Name | Size | Enrichment Score (ES) | Normalized Enrichment Score (NES) | Nominal P-Value | FDR Q-Value |
| POSITIONAL GENE SETS |  |  |  |  |  |
| CHR 7q21 | 5 | -0.68 | -1.7 | 0.015 | 0.148 |
| GENE ONTOLOGY - BIOLOGICAL PROCESSES |  |  |  |  |  |
| Negative Regulation of Cellular Metabolic Process | 12 | 0.51 | 2.29 | 0 | 0.043 |
| Negative Regulation of Metabolic Process | 12 | 0.51 | 2.21 | 0 | 0.046 |
| Homeostatic Process | 10 | 0.56 | 2.2 | 0.004 | 0.032 |
| Chemical Homeostasis | 7 | 0.56 | 1.89 | 0.018 | 0.154 |
| Interphase | 5 | 0.67 | 1.88 | 0.006 | 0.129 |
| Interphase of Mitotic Cell Cycle | 5 | 0.67 | 1.87 | 0.024 | 0.121 |
| Regulation of Biological Quality | 21 | 0.32 | 1.78 | 0.032 | 0.166 |
| Cell Fate Commitment | 5 | 0.62 | 1.73 | 0.037 | 0.184 |
| GENE ONTOLOGY - MOLECULAR FUNCTION |  |  |  |  |  |
| Purine Ribonucleotide Binding | 11 | 0.48 | 1.98 | 0 | 0.192 |
| Purine Nucleotide Binding | 11 | 0.48 | 1.97 | 0.013 | 0.098 |
| Nucleotide Binding | 11 | 0.48 | 1.96 | 0.012 | 0.071 |
| ATP Binding | 10 | 0.47 | 1.83 | 0.007 | 0.122 |
| Adenyl Nucleotide Binding | 10 | 0.47 | 1.83 | 0.011 | 0.098 |
| Adenyl Ribonucleotide Binding | 10 | 0.47 | 1.82 | 0.012 | 0.082 |
| Kinase Activity | 29 | 0.27 | 1.81 | 0.028 | 0.073 |
| Transferase Activity - Transferring Phosphorus Containing Groups | 29 | 0.27 | 1.72 | 0.03 | 0.098 |
| Protein Kinase Activity | 28 | 0.26 | 1.71 | 0.027 | 0.095 |
| Magnesium Ion Binding | 5 | 0.62 | 1.69 | 0.024 | 0.09 |
| Phosphotransferase Activity - Alcohol Group as Acceptor | 28 | 0.26 | 1.62 | 0.036 | 0.114 |
| Protein Serine Threonine Kinase Activity | 14 | 0.34 | 1.59 | 0.034 | 0.122 |
